# Post-COVID-19 vaccine Guillain-Barré syndrome; first reported case from Qatar

**DOI:** 10.1016/j.amsu.2021.102540

**Published:** 2021-07-03

**Authors:** Almurtada Razok, Abdullah Shams, Ahmed Almeer, Muhammad Zahid

**Affiliations:** Department of Internal Medicine, Hamad Medical Corporation, P.O 3050, Doha, Qatar

**Keywords:** COVID-19 vaccine, Guillain-Barré syndrome, Molecular mimicry, Qatar

## Abstract

**Introduction:**

Guillain-Barré syndrome (GBS) is an immune-mediated peripheral neuropathy that was reported following meningococcus, polio, influenza and rabies vaccines. However, an association with the COVID-19 vaccine is yet to be established.

**Presentation of case:**

We present the case of an elderly gentleman with no history of SARS-CoV-2 infection or any recent viral or bacterial illnesses who presented with GBS 20 days after the second dose of COVID-19 vaccination. The diagnosis was established based on physical examination, magnetic resonance imaging (MRI) of the spine, cerebrospinal fluid (CSF) analysis and electromyography (EMG).

**Discussion:**

Due to the occurrence of GBS after certain types of infections, molecular mimicry has become widely acceptable as the underlying pathophysiology. The reported cases of GBS following vaccination further supported this theory, however proving a causal relationship between vaccines and GBS on the molecular level remains a challenge.

**Conclusions:**

To the best of our knowledge this is the first reported case in the state of Qatar. It is important to mention that more research is needed to establish an association between COVID-19 vaccine and GBS. In our opinion, the benefits of COIVID-19 vaccine largely outweigh its risks.

## Highlights

• COVID-19 vaccines and their adverse events are an emerging topic in medicine.
• GBS was reported following influenza, hepatitis A and COVID-19 vaccines.
• It is an immune-mediated peripheral neuropathy triggered by molecular mimicry.
• More research is needed to confirm an association with COVID-19 vaccine.

## 1 Introduction

Guillain–Barré syndrome (GBS) is an immune-mediated disorder with an estimated annual incidence of 1–2 cases per 100,000 worldwide. It is the most common cause of acute non-trauma related paralysis in the developed world [1,2]. It manifests as acute, rapidly progressing polyradiculoneuropathy due to inflammation and demyelination of the peripheral nervous system, resulting in a classically symmetrical and ascending weakness, often in association with hyporeflexia or areflexia [3]. The exact cause of Guillain–Barre syndrome is still unknown, but the suggested pathophysiology is molecular mimicry following respiratory and gastrointestinal infections.

After the first case was reported in Wuhan, China in December 2019, the global pandemic caused by SARS-CoV-2 brought many challenges including the manufacturing and administration of vaccine. Several vaccines were approved by FDA and reported side effects ranged from pain at the site of injection, myalgia, fatigue, and fever to more serious ones including anaphylaxis [4,5]. GBS was historically linked with some vaccines namely, rabies, hepatitis A and B, polio and influenza [6].

We report this case to increase awareness on GBS as a possible complication of COVID-19 vaccine and to help evolving studies understand if the syndrome is associated with a specific type of the vaccine or is more likely to occur in a specific patient population.

## 2 Presentation of case

A 73-year-old gentleman, active smoker, with medical history of hypertension and well-controlled rheumatoid arthritis, presented to the emergency department through ambulance service with 3–4 days’ complaints of progressive bilateral lower limb weakness which prevented him from carrying out his activities of daily living. He previously had an excellent functional status and denied any history of recent trauma, fever, upper respiratory or gastrointestinal tract illness. There was no weight loss, night sweats or change in bowel habits. He had received the second dose of COVID-19 vaccine (Pfizer) 20 days prior to his presentation.

On physical examination, the patient was vitally stable, afebrile and on room air with no signs of distress. Neurological examination showed intact sensation in both upper and lower limbs. Motor strength according to Medical Research Council grade was 5/5 in upper limbs and 3/5 in both lower limbs, proximally and distally. The patient was not able to walk or maintain sitting posture on his own. His reflexes were absent in the ankles, reduced in the knees bilaterally (2/4), and normal in the upper limbs. There was no nystagmus or dysdiadochokinesia. Examination of the cranial nerves and other systems was normal.

**Fig. 1:**
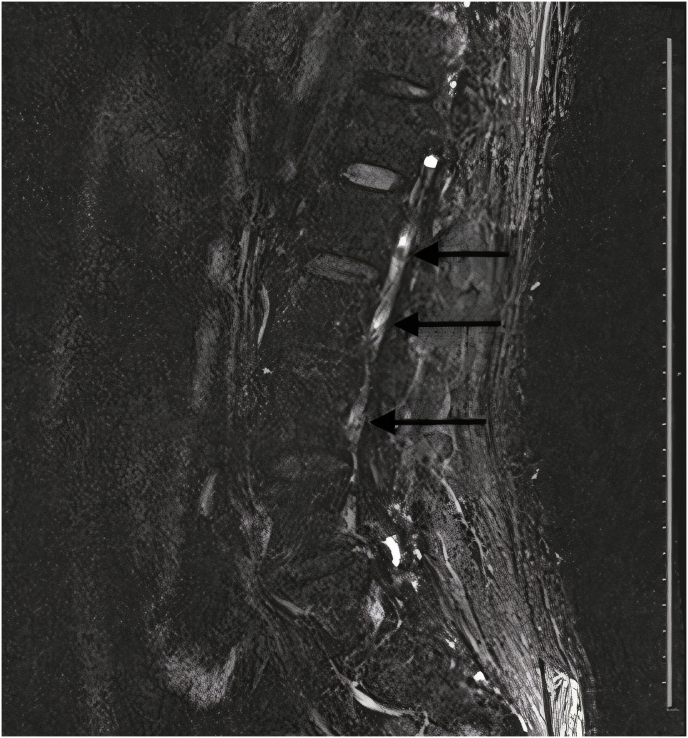
Magnetic resonance image of the spine with intravenous gadolinium showing bilateral contrast enhancement of the nerve roots in the lumbar region and the upper part of the cauda equina (as indicated by the arrows).

Complete blood count showed mild leukocytosis of 11.9 × 10^3^/μL (reference range 4–10 × 10^3^/μL) with neutrophilic predominance, and normal hemoglobin and platelet count. His renal, hepatic and coagulation profile were normal. C-reactive protein was elevated at 54 mg/dl (reference range 0–5 mg/dl). COVID-19 PCR from a nasopharyngeal swab was negative. Computed tomography (CT) and magnetic resonance imaging of the brain were negative for any acute insult in the cerebellum and brainstem. A lumbar puncture was performed, and cerebrospinal fluid analysis showed normal glucose in addition to normal white and red blood cell counts. Additionally, CSF analysis showed elevated protein at 0.8 gm/L (reference range 0.15–0.45 gm/L) and elevated albumin at 421 mg/L (reference range 0–350 mg/L). Gram stain and culture of the fluid were negative. Oligoclonal band from the CSF was negative as well. MRI of the spine showed bilateral nerve root enhancement in the lumbar region and the upper part of the cauda equina (Fig. 1).

Nerve conduction study (NCS) and electromyogram (EMG) showed bilateral absent H reflexes in the gastrocnemius muscles consistent with early polyneuroradiculopathy.

Based on the previous work up a diagnosis of GBS was made. The patient had a stable forced vital capacity (FVC) above 80% throughout his stay in the hospital. He received human intravenous immunoglobulin (IVIG) at a dose of 0.4 gm/kg/day for five days, after which he showed signs of improvement in ambulation and overall function. He tolerated the IVIG without experiencing any side effects. As he remained stable and responded well to IVIG, he was transferred to an inpatient rehabilitation facility 12 days after admission, where he received extensive physical and occupational therapy for two months’ duration. He was seen in the neurology clinic, outpatient department of the hospital three weeks after discharge from the rehabilitation institute, where physical examination showed full recovery of the muscle power (grade 5/5) and functional status (Assessed using the Medical Research Council grade).

**Patient perspective**: “Although I had to go through multiple investigations, I was pleased that the medical team was able to identify my diagnosis and provide me with the appropriate treatment. I was wondering all the time if what I had was triggered by the vaccine which I recently received, I hope this will be clarified soon”.

## 3 Discussion

Since the widespread use of COVID-19 vaccines, healthcare practitioners and authorities had a special and justified interest in the side-effects and complications associated with these vaccines. The commonly reported side-effects include both local and systemic manifestations, in addition to asymptomatic laboratory abnormalities. Local side effects which developed at the site of administration include pain, swelling and erythema. Systemic ones include febrile reaction, muscle pain and fatiguability. Laboratory abnormalities include deranged transaminases, hyperbilirubinemia, and anemia [7]. Of more concern were the less common, yet more serious complications, which include myocarditis and thrombosis. When it comes to myocarditis, there seems to be a predominance of males and young age [8]. However, Vaccine‐associated Immune Thrombosis and Thrombocytopenia (VITT) syndrome did not share the same trait. Of note, there was a predilection for intracerebral thrombosis. The underlying pathophysiology was immune-mediated and resembled the one behind heparin-induced thrombocytopenia (HIT) [9].

Guillain-Barré syndrome (GBS) is also immune-mediated and encompasses a variety of demyelinating conditions which include acute inflammatory demyelinating polyradiculoneuropathy (AIDP), acute motor axonal neuropathy, acute motor-sensory axonal neuropathy, and Miller Fisher syndrome [10]. The annual incidence of GBS in the United States has been estimated as 1.65–1.79 case per 100,000 [11]. The incidence of GBS seems to increase with advancing age and is higher in males than females with a ratio of 1.5:1. GBS has become one of the leading non-traumatic causes of acute flaccid paralysis (AFP), especially in developed countries. The classical presentation of GBS is bilateral symmetric weakness and decreased deep tendon reflexes with or without accompanying sensory symptoms such as numbness or tingling. The most helpful investigations include a lumbar puncture with CSF analysis demonstrating albuminocytological dissociation and electrophysiological studies showing peripheral neuropathy which is either demyelinating or axonal in origin [12]. The post-infectious occurrence of GBS led to the reinforcement of the molecular mimicry theory as the underlying pathophysiology. It was postulated that certain infectious agents such as *Campylobacter jejuni* can lead to the formation of cross-reactive antibodies that target gangliosides which constitute a part of the myelin sheath encircling the peripheral nerves [13]. The axonal neuropathy observed in rabbits following their injection with ganglioside-like structures extracted from the bacterial cell wall of *C.jejuni* further support this theory [14].

The immunological pathogenesis of GBS was further reinforced by the reported cases following vaccination against multiple pathogens. The influenza vaccine was the most notorious, however others such as hepatitis A and tetanus were also on the list of possibly associated vaccines [15,16]. Despite a relatively large number of reported cases of post-vaccination GBS, a definite causal association was not confirmed. The increased cases of GBS following the administration of the swine influenza vaccine between 1967 and 1977 did point towards a possible causality, however the results generated by further studies conducted years after were less conclusive. The same applied for the oral polio and tetanus vaccines. Earlier studies suggested possible causality; however, the results were contradicted by epidemiological studies performed later [17].

The first case of GBS following COVID-19 vaccination was reported in February 2021 in the USA in an elderly female who presented 2 weeks after the first dose of the vaccine. The patient presented with fatigue and bilateral symmetric weakness of the lower limbs. CSF analysis showed albuminocytological dissociation and she was started on IVIG which led to improvement in the weakness. The patient recovered successfully and was discharged to a rehabilitation institute thereafter [18].

Considering the uncertainty of the causal relation between vaccines and GBS and the inability to prove that relation on a molecular basis, a temporal association is a possibility. However further studies are required before establishing a conclusion. We would like to express our opinion that the reduction in morbidity and mortality achieved by vaccination outweighs the risks of the reported adverse events and extensive research is required before asserting or ruling out a causal relation between COVID-19 vaccine and GBS.

## 4 Conclusion and learning points

With the increased reports of GBS following COVID-19 vaccine, the Therapeutic Goods Administration (TGA) declared GBS “an adverse event of special interest”. We hope that our case will serve as a bridge to further research on this subject and will alarm healthcare workers to consider GBS as a diagnosis in patients who present with acute flaccid paralysis (AFP) after receiving the COVID-19 vaccine.

This work was reported in line with the CARE guidelines.

## Consent for publication

Written informed consent was obtained from the patient for publication of this case report and the accompanying image. A copy of the written consent is available for review by the Editor-in-Chief of this journal on request.

## Ethical approval

Written informed consent was obtained from the patient for publication of this case report and the accompanying image. A copy of the written consent is available for review by the Editor-in-Chief of this journal on request.

## Availability of data and materials

The datasets used and/or analyzed during the current study are available from the corresponding author on reasonable request.

## Funding statement

This article did not receive any specific grant from funding agencies in the public, commercial, or not-for-profit sectors.

## Author contribution

AR and AS performed literature review and wrote the original draft of the manuscript. AA and MZ supervised the writing process and revised the manuscript. All authors approved the final version for submission.

## Research registration

Non-applicable.

## Provenance and peer review

Not commissioned, externally peer-reviewed.

## Guarantor

Almurtada Razok.

## Declaration of competing interest

None to be declared.

## References

[1] Agha Riaz A., Borrelli Mimi R., Farwana Reem, Koshy Kiron, Fowler Alexander J., Orgill Dennis P. (2018). For the SCARE Group. The SCARE 2018 statement: updating consensus Surgical CAse REport (SCARE) guidelines. Int. J. Surg..

[2] Sejvar J.J., Baughman A.L., Wise M., Morgan O.W. (2011). Population incidence of Guillain-Barré syndrome: a systematic review and meta-analysis. Neuroepidemiology.

[3] Willison H.J., Jacobs B.C., van Doorn P.A. (2016 Aug 13). Guillain-Barré syndrome. Lancet.

[4] Kim J.H., Marks F., Clemens J.D. (2021 Feb). Looking beyond COVID-19 vaccine phase 3 trials. Nat. Med..

[5] Polack F.P., Thomas S.J., Kitchin N., Absalon J., Gurtman A., Lockhart S., Perez J.L., Pérez Marc G., Moreira E.D., Zerbini C., Bailey R., Swanson K.A., Roychoudhury S., Koury K., Li P., Kalina W.V., Cooper D., Frenck R.W., Hammitt L.L., Türeci Ö., Nell H., Schaefer A., Ünal S., Tresnan D.B., Mather S., Dormitzer P.R., Şahin U., Jansen K.U., Gruber W.C. (2020 Dec 31). C4591001 clinical trial group. Safety and efficacy of the BNT162b2 mRNA covid-19 vaccine. N. Engl. J. Med..

[6] Schonberger L.B., Bregman D.J., Sullivan-Bolyai J.Z., Keenlyside R.A., Ziegler D.W., Retailliau H.F., Eddins D.L., Bryan J.A. (1979 Aug). Guillain-barre syndrome following vaccination in the national influenza immunization program, United States, 1976--1977. Am. J. Epidemiol..

[7] Kaur R.J., Dutta S., Bhardwaj P., Charan J., Dhingra S., Mitra P., Singh K., Yadav D., Sharma P., Misra S. (2021 Mar 27). Adverse events reported from COVID-19 vaccine trials: a systematic review. Indian J. Clin. Biochem..

[8] Abu Mouch S., Roguin A., Hellou E., Ishai A., Shoshan U., Mahamid L., Zoabi M., Aisman M., Goldschmid N., Berar Yanay N. (2021 Jun 29). Myocarditis following COVID-19 mRNA vaccination. Vaccine.

[9] Franchini M., Liumbruno G.M., Pezzo M. (2021 May 13). COVID-19 vaccine-associated immune thrombosis and thrombocytopenia (VITT): diagnostic and therapeutic recommendations for a new syndrome. Eur. J. Haematol..

[10] Walling A.D., Dickson G. (2013 Feb 1). Guillain-Barré syndrome. Am. Fam. Physician.

[11] Alshekhlee A., Hussain Z., Sultan B., Katirji B. (2008 Apr 29). Guillain-Barré syndrome: incidence and mortality rates in US hospitals. Neurology.

[12] Bogliun G., Beghi E., Italian GBS Registry Study Group (2004 Aug). Incidence and clinical features of acute inflammatory polyradiculoneuropathy in Lombardy, Italy, 1996. Acta Neurol. Scand..

[13] van Doorn P.A., Ruts L., Jacobs B.C. (2008 Oct). Clinical features, pathogenesis, and treatment of Guillain-Barré syndrome. Lancet Neurol..

[14] Hughes R.A., Cornblath D.R. (2005 Nov 5). Guillain-Barré syndrome. Lancet.

[15] Souayah N., Nasar A., Suri M.F., Qureshi A.I. (2007 Jul 20). Guillain-barre syndrome after vaccination in United States a report from the CDC/FDA vaccine adverse event reporting system. Vaccine.

[16] Tuttle J., Chen R.T., Rantala H., Cherry J.D., Rhodes P.H., Hadler S. (1997 Dec). The risk of Guillain-Barré syndrome after tetanus-toxoid-containing vaccines in adults and children in the United States. Am. J. Publ. Health.

[17] Haber P., Sejvar J., Mikaeloff Y., DeStefano F. (2009). Vaccines and guillain-barré syndrome. Drug Saf..

[18] Waheed S., Bayas A., Hindi F., Rizvi Z., Espinosa P.S. (2021 Feb 18). Neurological complications of COVID-19: guillain-barre syndrome following pfizer COVID-19 vaccine. Cureus.

